# pH-Responsive Micelles Assembled by Three-Armed Degradable Block Copolymers with a Cholic Acid Core for Drug Controlled-Release

**DOI:** 10.3390/polym11030511

**Published:** 2019-03-18

**Authors:** Jingjie Feng, Weiqiu Wen, Yong-Guang Jia, Sa Liu, Jianwei Guo

**Affiliations:** 1 School of Chemical Engineering & Light Industry, Guangdong University of Technology, Guangzhou 510006, China; fjj121036@163.com (J.F.); 15024028760@163.com (W.W.); 2 School of Materials Science and Engineering, South China University of Technology, Guangzhou 510641, China; ygjia@scut.edu.cn

**Keywords:** cholic acid, pH-sensitive, PTX, drug delivery

## Abstract

One of the most famous anticancer drugs, paclitaxel (PTX), has often been used in drug controlled-release studies. The polymers derived from bio-compound bile acids and degradable poly(ε-caprolactone) (PCL) form a reservoir and have been used as a drug delivery system with great advantages. Herein, we grafted poly(*N*,*N*-diethylaminoethyl methacrylate) and poly(poly(ethylene glycol) methyl ether methacrylate) into the bile acid-derived three-armed macroinitiator CA-(PCL)_3_, resulting in the amphiphilic block copolymers CA-(PCL-*b*-PDEAEMA-*b*-PPEGMA)_3_. These pH-responsive three-armed block copolymers self-assembled into micelles in aqueous solution and PTX was encapsulated into the micellar core to form PTX-loaded micelles with a drug loading of 29.92 wt %. The micelles were stable in PBS at pH 7.4 and showed a pH-triggered release behavior of PTX under acidic environments, in which 55% of PTX was released at pH 5.0 in 80 h. These cholic acid-based functionalized three-armed block polymers present good biocompatibility, showing great potential for drug controlled-release.

## 1. Introduction

According to the latest report released by the International Agency for Research on Cancer (IARC), the number of cancer deaths in 2018 has reached 9.6 million, which is increasing year by year, with cancer becoming one of the major causes of mortality in the world. Over the past several decades, chemotherapy has been considered as one of the efficient methods for cancer therapy and has gained unprecedented attention. It has been demonstrated that typically, as a kind of anticancer agent, paclitaxel (PTX) and its derivatives such as Abraxane^®^, Taxotere^®^ and Taxol^®^, to name a few, have a certain therapeutic efficacy on the treatment of ovarian cancer [1], breast cancer [2] as well as lung cancer [3]. To date, these series of anticancer drugs still face several limitations, including poor water solubility and uncontrollable pharmacokinetic processes. Furthermore, the nonspecific biodistribution of the anticancer drugs usually causes severe side effects on normal tissue and undermines the therapy effect on tumor tissue [4,5]. In order to effectively improve the therapeutic efficacy as well as minimize the side effects mentioned above, an increasing attention has been paid to the development of a drug delivery system, including liposomes [6,7], micelles [8], vesicles [9,10], mesoporous silica nanoparticles [11], and other nanoparticles.

Notably, polymeric micelles are the preferred drug delivery system for anticancer drugs because of their unique structure, adjustable size and shape, simple synthesis route, and tailor-made functionalization [12,13,14]. As is well known, polymeric micelles with a unique core-shell structure are spontaneously assembled from amphiphilic block copolymers, and the micelle inner core that is made up of hydrophobic polymer chains serves as a nanocontainer for the loading of hydrophobic anticancer drugs [15]. However, a drug controlled-release system of polymer-based micelles still faces several challenges, namely, the burst release of anticancer drugs, poor biodegradability, and target-free delivery of tumor tissue [16,17,18].

Interestingly, compared with normal tissues, tumor tissues have some differences in the tissue microenvironment, such as weak acidity [19], abnormal temperature gradients [20,21], overexpressed proteins and enzymes, as well as a higher concentration of glutathione (GSH) or cysteine [22,23]. Based on these significant differences in microenvironment, a series of stimulus-responsive polymeric micelles have been exploited to control the release of the anticancer drugs at the expected target sites. For example, researchers used the weak acidity of tumor tissues and developed a series of pH-responsive drug controlled-release systems, which can accept appropriate pH changes in response to acidic fluctuation of the tumor tissues [24,25,26].

Cholic acid (CA), a naturally occurring bile acid, is derived from liver cells in the human body. Due to its special structure, cholic acid and its derivatives have attracted the attention of researchers, and their application in drug controlled-release systems has been reported extensively [27,28,29,30]. Zhu and co-workers reported the synthesis of series polymers based on CA, and using them in drug delivery systems. Research indicated that the micelles formed from cholic acid-based star polymers had a lower density than linear polymers with a similar structure, forming an internal reservoir that can accommodate a larger amount of hydrophobic therapeutic drug, showing their advantages in terms of biocompatibility and drug loading [31,32,33,34,35]. The drug loading and encapsulation efficiency of these nanoparticles reached up to 35 wt % and higher than 89%, respectively, improving bioavailability and pharmacokinetics of the drug.

**Figure 1:**
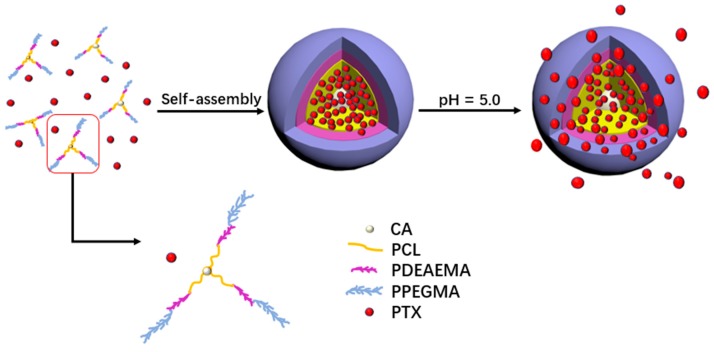
Schematic diagram of self-assembly and pH response release of polymer drug-loaded micelles.

**Scheme 1:**
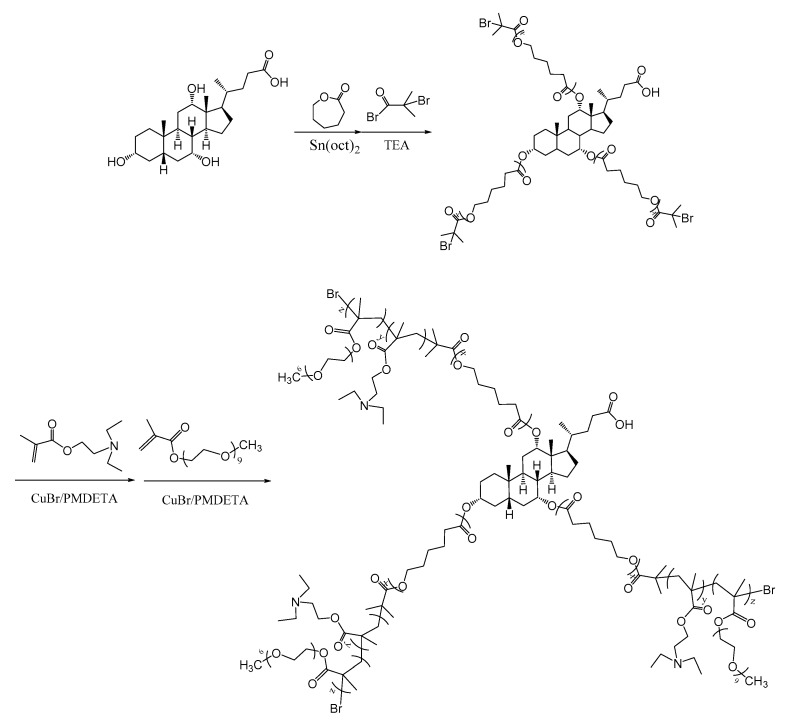
Synthesis of the block polymer CA-(PCL-*b*-PDEAEMA-*b*-PPEGMA)_3_.

Herein, biocompatible hydrophobic poly(ε-caprolactone) (PCL) was bonded with cholic acid by ring-opening polymerization (ROP) to form the micellar core during the self-assembly. Then pH-responsive block poly (2-(diethylamino) ethyl methacrylate) and hydrophilic block poly (ethylene glycol) methyl acrylate (PPEGMA) were sequentially grafted onto PCL via atom transfer radical polymerization (ATRP) to obtain a three-armed star amphiphilic block polymer, CA-(PCL-*b*-PDEAEMA-*b*-PPEGMA)_3_, also named as CA-CDEP (Scheme 1). For comparison, CA-(PCL-*b*-PPEGMA)_3_, also named as CA-CP, without pH-responsive block was also synthesized under the same conditions (Scheme S1). PTX was encapsulated in these two types of polymeric micelles. The drug was expected to release in a weakly acidic environment due to the swelling of micelles in the presence of block PDEAEMA (Figure 1). Furthermore, the self-assembly, drug loading performance, drug release, and in vitro cytotoxicity of these polymeric micelles were also evaluated.

## 2. Materials and Methods

### 2.1. Materials

All chemical reagents were used as received unless otherwise noted. Cholic acid (CA, 98%), 2-bromoisobutyryl bromide (2-BIBB, 98%), copper(I) bromide (CuBr, 99%), *N*,*N*,*N*′,*N*′,*N*″-pentamethyl diethylene-triamine (PMDETA, 98%), 2-(diethylamino) ethyl methacrylate (DEAEMA, 99%), poly (ethylene glycol) methyl ether methacrylate (PEGMA, M_n_ = 500 Da) and paclitaxel (PTX, 99%) were purchased from Aladdin (Shanghai, China). ε-caprolactone (ε-CL, 99%) and stannous octoate (S_n_(Oct)_2_, 95%) were purchased from Macklin Biochemical Co. Ltd. (Shanghai, China). Dichloromethane (DCM, AR), triethylamine (TEA, AR), tetrahydrofuran (THF, AR), dimethyl sulfoxide (DMSO, AR), and dimethyl formamide (DMF, AR) were purchased from Energy Chemical (Shanghai, China). All other solvents such as ethyl alcohol were supplied by Guangzhou chemical reagent factory and used directly. Copper(I) bromide was purified via washing with acetic acid and ethyl alcohol three times, respectively, and then stored in a vacuum glove box. DEAEMA and PEGMA were purified by column-chromatography over basic alumina to remove the inhibitors before used. TEA, DCM, THF, and ε-CL were stirred overnight with CaH_2_ at room temperature and distilled at atmospheric pressure to remove any water remaining in the solvent, then stored with 4A molecular sieves.

### 2.2. Characterization

^1^H NMR spectra were recorded on a Bruker AVANCE III 400 MHz superconducting Fourier (Bruker, Billerica, MA, USA) in deuterium generation reagent with tetramethyl silane (TMS) as the internal standard for structure characterization of polymers. The number average molecular weight (M_n_) was detected by gel permeation chromatography (GPC) (Waters 1515/2414, Waters, Milford, MA, USA), using THF as the mobile phase with a flow rate of 1.0 mL/min and polystyrene (PS) as the standard for calibration. The morphological characterization of the polymeric micelles was characterized with a HT7700 transmission electron microscopy (TEM, Hitachi, Japan). The sizes and zeta potential of the block polymers were measured by dynamic light scattering (DLS) with a Zeta PALS zeta potential and granularity analyzer (Brookhaven, New York, NY, USA). The fluorescence spectra were examined on a FluoroMax-4 fluorescence spectrometer (HORIBA Jobin Yvon, Clifton Park, NY, USA). UV–Vis spectra were performed on a UV2450 spectrophotometer (Shimadzu, Kyoto, Japan). The copper residues in the polymer were characterized by an Escalab 250Xi X-ray photoelectron spectroscopy (XPS, Thermo Fisher, West Sussex, UK).

### 2.3. Synthesis of Polymers

#### 2.3.1. Synthesis of CA-(PCL_28_)_3_

Cholic acid (0.4 g, 1 mmol) and ε-CL (6.8 g, 60 mmol) were added to a Schlenk flask and degassed with three freeze-pump-thaw cycles to remove oxygen. Then, Sn(Oct)_2_ (36 mg, 0.088 mmol) mixed with toluene (0.5 mL) was injected into the flask under inert atmosphere protection, followed by continuous stirring and heating at 160 °C for 8 h. After cooling down to room temperature, the crude product was dissolved in THF and the undissolved solid was removed by filtration. Subsequently, the collected filtrate was precipitated into excess cold n-hexane and further purification was performed by successively washing with ethyl alcohol and petroleum ether for three times. The purified polymer was dried at 40 °C in vacuum overnight to obtain the white powder.

#### 2.3.2. Synthesis of CA-(PCL_28_-Br)_3_

CA-(PCL_28_-Br)_3_ as the initiator was synthesized by bromination reaction with 2-BIBB. CA-(PCL_28_)_3_ (2.0 g, 0.21 mmol) and TEA (0.3 g, 3 mmol) were added into a dried flask, then dissolved in 25 mL DCM at 0 °C. After being degassed with three freeze-pump-thaw cycles, 2-BIBB (0.35 mL, 3 mmol) dissolved in anhydrous DCM (5 mL) was fed dropwise into the flask with vigorous stirring under ice bath conditions. The mixture was continually stirred at 0 °C for 2 h and then at room temperature for another 24 h. The sediment was removed by filtration and the filtrate were successively washed by HCl (1 mol/L), saturated NaHCO_3_, and deionized water. After drying over anhydrous magnesium sulfate, the product was precipitated into excess n-hexane and then collected by drying at 40 °C in vacuum overnight.

#### 2.3.3. Synthesis of CA-(PCL_28_-*b*-PDEAEMA_5_)_3_

CA-(PCL_28_-*b*-PDEAEMA_5_)_3_ was prepared via ATRP. CA-(PCL_28_-Br)_3_ (1.0 g, 0.1 mmol), DEAEMA (1.1 g, 6 mmol), and PMDETA (36.4 mg, 0.21 mmol) were dissolved into 30 mL anhydrous THF and degassed with three freeze-pump-thaw cycles. Under the protection of argon, CuBr (20 mg, 0.139 mmol) as the catalyst was fed into the mixture and the system was degassed with three freeze-pump-thaw cycles again. The system was stirred at 65 °C for 24 h followed by the presence of oxygen to terminate the chain growth. The unreacted catalyst was removed by passing it through a neutral alumina column (such as THF as the eluent). The organic solution was concentrated by rotary evaporation, and then dispersed into ten times volume of n-hexane. Finally, the insoluble part was dried at 40 °C in vacuum for 24 h to obtain the pure product.

#### 2.3.4. Synthesis of CA-(PCL_28_-*b*-PDEAEMA_5_-*b*-PPEGMA_5_)_3_ (CA-CDEP)

As shown in Scheme 1, CA-(PCL_28_-*b*-PDEAEMA_5_)_3_ (1.5 g, 0.11 mmol) as the initiator with hydrophilic monomer PEGMA (1.0 g, 2 mmol) and PMDETA (36.4 mg, 0.21 mmol) as the ligand were dissolved into 20 mL anhydrous THF and degassed with freeze-pump-thaw cycle three times to eliminate the extra oxygen. Then, CuBr (20 mg, 0.139 mmol) was quickly added into the Schlenk flask and degassed with three freeze-pump-thaw cycles to make sure the system was keeping an oxygen-free condition to remain the active state of catalyst. The polymerization was conducted at 65 °C for 24 h in the oil bath. The mixture was diluted with THF and passed through a neutral alumina column to remove the catalysts. The organic solution was concentrated by removing eluent from rotary evaporation, and then the polymer was precipitated from an excess of hexane. The pure product CA-(PCL_28_-*b*-PDEAEMA_5_-*b*-PPEGMA_5_)_3_, named as CA-CDEP, was obtained after being dried in a vacuum at 40 °C for 24 h.

#### 2.3.5. Synthesis of CA-(PCL_28_-*b*-PPEGMA_7_)_3_ (CA-CP)

CA-(PCL_28_-*b*-PPEGMA_7_)_3_ was prepared via a similar method using CA-(PCL_28_-Br)_3_ as the macroinitiator. CA-(PCL_28_-Br)_3_ (1.0 g, 0.1 mmol), PEGMA (2.0 g, 4 mmol), and PMDETA (36.4 mg, 0.21 mmol) were dissolved into 30 mL anhydrous THF and degassed with three freeze-pump-thaw cycles. Under the protection of argon, CuBr (20 mg, 0.139 mmol) was fed into the solution as the catalyst and the whole system was degassed with three freeze-pump-thaw cycles. The system was stirred at 65 °C for 24 h. The catalyst was removed by passing it through a neutral alumina column using THF as the eluent. The organic solution was concentrated by rotary evaporation then dispersed into ten times volume of n-hexane. The product CA-(PCL_28_-*b*-PPEGMA_7_)_3_, named as CA-CP (Scheme S1), was collected by drying at 40 °C in vacuum for 24 h after filtration.

### 2.4. Self-Assembly of the Micelles

Briefly, the polymer (20 mg) was dissolved in 2 mL acetone, then added dropwise into the deionized water with a syringe and stirred at ambient temperature for 24 h. An initial polymer solution at a concentration of 1 mg/mL was obtained after removing the acetone. For the preparation of PTX-loaded micelles, the block polymer and PTX were dissolved into DMSO together. After being added dropwise into phosphate buffer (pH 7.4) with a syringe and stirred for 2 h, they were placed in a dialysis bag (MWCO = 3500 Da) and dialyzed against PBS solution for 6 h, then dialyzed against deionized water for 48 h. The solution was filtered through a 0.45 µm filter and kept in freeze-dried storage for further characterization.

### 2.5. CMC of the Micelles

The critical micelle concentration (CMC) of CA-CDEP and CA-CP were determined by a fluorescence spectrometer using pyrene as the fluorescence probe [36]. The fluorescence scanning range was from 300 to 350 nm and the emission wavelength was 373 nm. A quantity of pyrene was dissolved in 0.5 mL acetone, and then the acetone was volatilized overnight at room temperature in the dark. The initial polymer solution obtained above was diluted into a series of concentrations from 0.0001 to 0.1 mg/mL and fed into the vials containing pyrene. The mix solution was left to equilibrate in the dark for 24 h before being measured. The final concentration of pyrene in aqueous solution was 6.5 × 10^7^ M.

### 2.6. Drug Loading and In Vitro Release

PTX loading was measured by subtracting the amount of PTX released during quantitative purification from the amount of PTX in the feed. The amount of PTX in the micelles was determined by its absorbance at 258 nm on a UV–Vis spectrophotometer (UV2450), and combining with a calibration curve for PTX in DMSO. Then, 1 mg lyophilized PTX-loading micelles was dissolved into 10 mL DMSO, filtered using a 0.45 μm syringe filter.

The drug loading content (DLC) and entrapment efficiency (EE) were calculated based on three independent measurements as follows:

(1) $$DLC\;\left(\mathrm{wt}\;\%\right)=\frac{wt\;of\;loaded\;drug}{wt\;of\;drug-loaded\;micelle}\times 100\%,$$

(2) $$EE\;\left(\%\right)=\frac{wt\;of\;loaded\;drug}{wt\;of\;drug\;in\;feed}\times 100\%.$$

The PTX release behavior from PTX-loaded block polymer micelles was monitored by changing the pH of the ambient environment. First, 5 mg PTX-loaded micelles were dispersed in 5 mL PBS (1 mg/mL) and put into a dialysis bag (MWCO = 7000 Da), then immersed in PBS containing 1 wt % DMSO, thereby allowing us to better observe the release progress of the drug. The whole process was stirred in a shaking water bath at 37 °C. Different pH environments were provided by PBS and acetate buffer. An amount of 4 mL release medium was taken out at regular intervals and 4 mL fresh medium was replenished to maintain the same volume of the solution. The released amount of drug was determined using a UV–Vis spectrophotometer. The tests were repeated three times, and the results were presented as the average of three independent measurements.

### 2.7. In Vitro Cytotoxicity Assay

NIH-3T3 cells were incubated in 96-well plates at a density of 5000 cells per well with 80 μL culture medium (NIH-3T3 cells in 90% DMEM containing 10% FBS and 1% P/S), and the cytotoxicity was determined by a CCK-8 assay. The cells were incubated at 37 °C and 5% CO_2_ in medium for 24 h, then the culture medium was removed, and fresh medium with different concentrations of samples was added, the cells were incubated for a further 48 h. Free fresh culture medium was used as a control, and each sample was replicated in three cells. A 10 μL portion of Cell Counting Kit-8 solution was added to each well and the cells were incubated for 2 h. The absorbance of the solution was measured at 450 nm using a Nivo reader (PerkinElmer, Waltham, MA, USA). The experiments were repeated three times, and the results were obtained from three independent measurements. The cell viability was calculated as follows:

(3) $$\mathrm{Cell}\;\mathrm{viability}\;\left(\%\right)=\frac{OD_{S}}{AVER\;(OD_{nc})}\times 100\%,$$

where *OD_s_* is the absorption value of the sample wells and *OD_nc_* is the absorption value in the absence of samples. The background blank control well absorbance should be subtracted from the measured absorbance of each group.

## 3. Results and Discussion

### 3.1. Synthesis of Block Polymers

**Figure 2:**
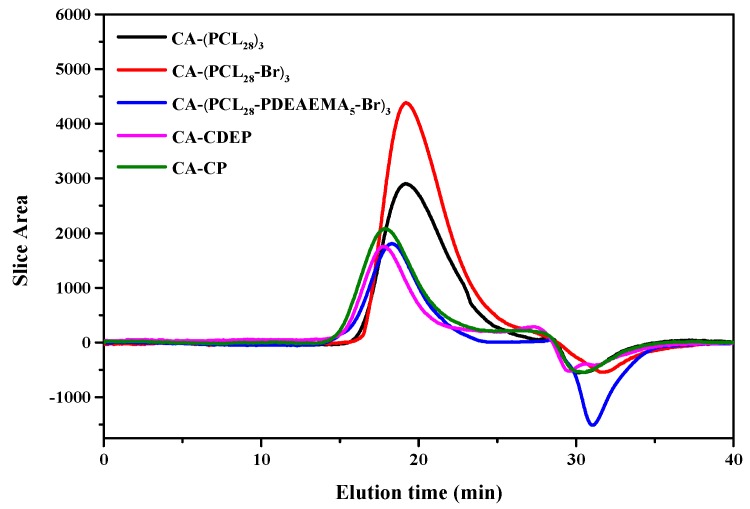
Gel permeation chromatography (GPC) analysis in THF of CA-(PCL_28_)_3_, CA-(PCL_28_-Br)_3_, CA-(PCL_28_-*b*-PDEAEMA_5_)_3_, CA-CDEP, CA-CP.

The amphiphilic block polymers CA-CDEP and CA-CP were both synthesized by a combination of ROP and ATRP. The molecular weight (M_n_) of all polymers was determined by GPC and the weights are summarized in Table 1. The GPC curves presented in Figure 2 exhibited unimodal symmetric distribution, indicating that ROP and ATRP processes were well controlled. As can be seen in Table 1, the M_n_ by GPC was lower than the M_n_ by theoretical calculation because of the relatively low conversion rate. The polydispersity indexes of overall polymers were between 1.30 and 1.55, which is beneficial for further drug delivery. The degrees of polymerization of ε-CL, DEAEMA, and PEGMA were approximately 84, 15, and 15, respectively, obtained from the GPC data. For CA-CP, the degrees of polymerization of ε-CL and PEGMA were about 84 and 21, respectively.

The ^1^H NMR spectra shown in Figures S1–S4 demonstrate the compositions of CA-CDEP and CA-CP. In Figure S1, the signals at 1.38, 1.65, 2.31, and 4.06 ppm were the characteristic peaks of –CH2– protons of PCL. The signal at 1.93 ppm was ascribed to –C(CH_3_)_2_–Br of CA-(PCL_28_-Br)_3_ in Figure S2. As presented in Figures S3 and S4, the peaks at 2.69 and 3.99 ppm were assigned as methylene protons of –CH_2_–CH_2_– in DEAEMA unit. The peaks at 2.58 and 1.02 ppm belonged to the methylene and end methyl protons of –CH_2_–CH_3_ in DEAEMA unit. The peaks at 0.81 and 1.75–1.90 ppm were ascribed to –CCH_3_ and –CH_2_– of the acrylate part. The chain extension of PPEGMA on CA-(PCL_28_-*b*-PDEAEMA_5_)_3_ results in the appearance of signals at 3.67 ppm (–OCH_2_–CH_2_O–), 3.38 ppm (–OCH_3_), and 4.23 ppm (–COO–CH_2_–).

### 3.2. Formation and Characterization of the Micelles

The amphiphilic block polymer consisting of hydrophobic PCL unit and hydrophilic PPEGMA unit self-assembled into micelles in aqueous solution. The CMC of polymeric micelle was an important parameter to determine the conformational states of polymer chains (single molecule or aggregate). The stability of the micelles in the human blood circulation became better due to the lower CMC value [37].

**Figure 3:**
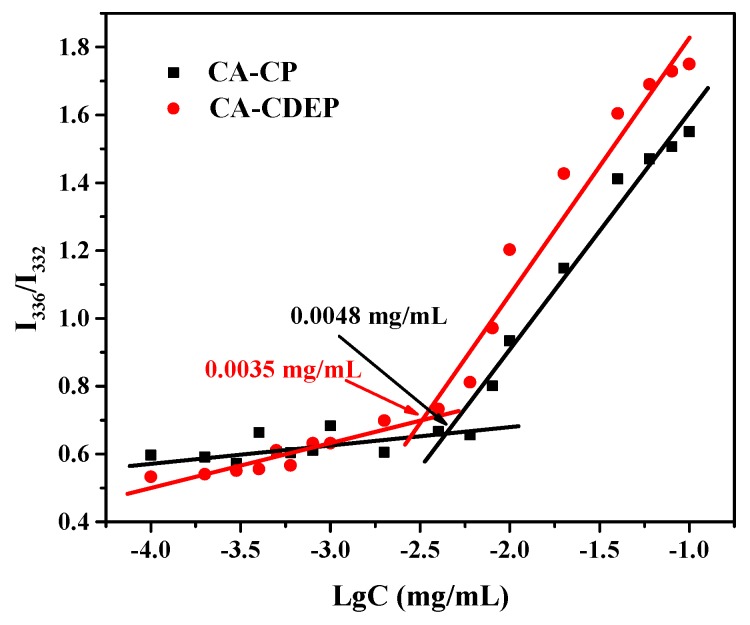
Intensity ratio (I_336_/I_332_) in the fluorescence spectra of pyrene as a function of polymeric concentrations in aqueous solution.

The CMC of polymeric micelles were performed on fluorescence spectroscopy with pyrene as fluorescence probe. As shown in Figure 3, the CMC value of CA-CDEP was 0.0035 mg/mL lower than that of CA-CP (0.0048 mg/mL). This may be attributed to the high hydrophobic portion of CA-CDEP with the presence of block PDEAEMA. In general, amphiphilic polymer micelles with low CMC values remain stable in the blood circulatory system and prevent chemotherapy drugs from releasing before reaching cancer cells [38].

Figure S5 shows the acid–base titration curve. During the continuous addition of NaOH, the pH value of polymer CA-CDEP presented a clear buffering trend, which ranged from 4.42 to 6.68. Such a response region was not observed for CA-CP, for which the variation trend was basically consistent with the NaCl solution. The results indicated that the pK_b_ of the CA-CDEP shifted to 5.53 after the introduction of the DEAEMA monomer, which is also the inflection point of the curve of the block polymer CA-CDEP.

**Figure 4:**
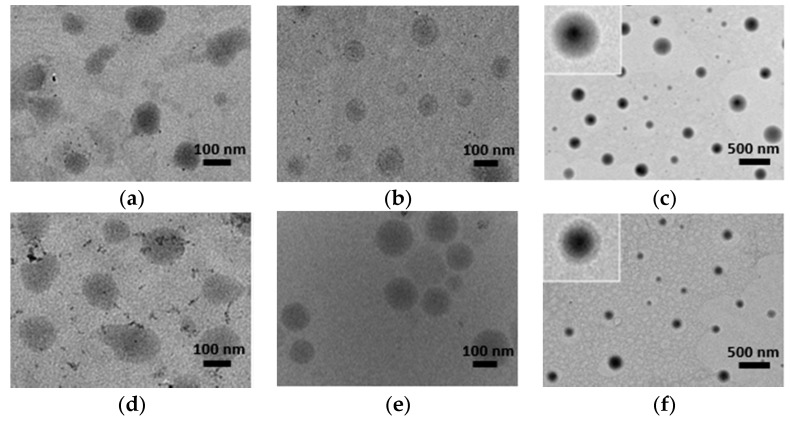
TEM images of the two polymers. The upper images show (**a**) CA-CP blank micelles at pH 7.4, (**b**) pH 5.0, and (**c**) PTX-loaded micelles. The lower images show (**d**) CA-CDEP blank micelles at pH 7.4, (**e**) pH 5.0, and (**f**) PTX-loaded micelles. The concentration of all samples is 0.5 mg/mL.

Spherical nanoparticles with a micellar structure were expected since the molecular mass of the hydrophobic block was less than that of the hydrophilic block [23,39]. The size changes of the amphiphilic block polymer micelles and PTX-loading micelles were observed by TEM and DLS. Figure 4 shows the TEM image of the polymer micelles CA-CP and CA-CDEP. Figure 4a,b shows the particle sizes of CA-CP blank micelles at pH 7.4 and 5.0, respectively, and both are around 100–120 nm. The particle size of PTX-loading CA-CP micelles was increased to 180–250 nm with the clear core-shell structure (Figure 4c). The blank micelles assembled by CA-CDEP showed a spherical structure with a diameter of 90–120 nm at pH 7.4 (Figure 4d). The size of these blank polymer micelles increased to about 140 nm when the pH was adjusted to 5.0 (Figure 4e). Such an increase in size may be caused by the electrostatic repulsion of the protonated tertiary amino group of the PDEAEMA units under acidic conditions [24]. From the TEM images of PTX-loading CA-CDEP micelles (Figure 4f), the size increased from approximately 120 nm to approximately 230 nm compared with the blank micelles. The larger diameter of the drug-loaded micelles may be due to the entrapment of the PTX causing the hydrophobic layer to swell. Meanwhile, the spherical core-shell structure became more obvious after drug loading from the TEM image, which also proves that the drug is well wrapped in the polymer micelles.

**Figure 5:**
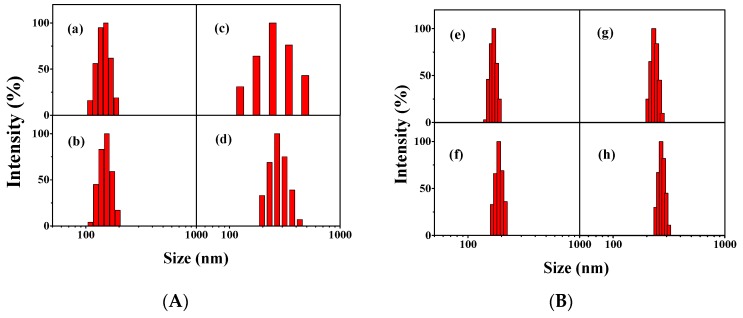
Particle-size distribution of micelles in PBS. (**A**) CA-CP, (**B**) CA-CDEP; (**a**,**e**) for blank micelles at 7.4; (**b**,**f**) for blank micelles at 5.0; (**c**,**g**) for drug-loaded micelles 3/10; (**d**,**h**) for drug-loaded micelles 5/10.

It is known that the size of micelles between the aqueous dispersions and the dry state is somewhat different. The mean particle sizes of the CA-CP micelles, CA-CDEP micelles, PTX-loaded CA-CP micelles, and PTX-loaded CA-CDEP micelles were 151.0, 170.4, 269.2, and 268.2 nm measured by DLS, at pH 7.4, respectively, which is a little larger than that observed from TEM (Figure 5). This may be due to the hydration of the PPEGMA chains in the aqueous medium [40]. The effect of pH on the morphological changes was also studied by DLS. Figure S6 shows the size of micelles at different pH ranging from 2 to 10. When the pH was adjusted from 10 to 7, there was no significant effect on the size of the micelles. The size of CA-CDEP micelles increased to 188 nm when pH was dropped from 7 to 4, because the tertiary amine group in the PDEAEMA segment was gradually protonated under the acidic conditions and the micelles expanded to balance the increasing electrostatic repulsion. The size decreased as the pH was further lowered from 4 to 2, which demonstrated that the electrostatic repulsion was greater than the hydrophobic interaction within the micelle, thus the number of polymer aggregates decreased [15,41]. In contrast, for the CA-CP micelles without the DEAEMA group, the size remained basically unchanged.

Figure S7 presents the zeta potential of both micelles when the pH was adjusted from 10 to 2. In an acidic environment, the charge of the micelles was positive, which could enhance the enhanced permeability and retention (EPR) effect of micelles for a longer time [42]. The zeta potential of micelles was negative at the alkaline environment, probably due to the hydrolysis of esters in the alkaline media. Overall, the introduction of DEAEMA increased the pH responsiveness of the polymer micelles, providing the possibility for the drug controlled-release.

### 3.3. Drug Release Assay

The PTX-loading and release properties of micelles were investigated using the UV–Vis method. Two different masses of PTX were loaded into the micelles, where the feed ratios of PTX to polymer were 3/10 and 5/10, respectively. As listed in Table 2, the DLC of the polymer micelles was mainly affected by the polymer chemical structures and drug-loading feed. The DLC of PTX-loaded micelles increased as the drug-loading feed increased. At the same dose, the polymer micelles CA-CDEP with longer length of PCL and PDEAEMA block displayed a higher drug loading capacity. DLC of CA-CDEP (29.92%) at a feed ratio of 5/10 was more than twice that of CA-CP micelles (11.4%). Clearly, the increase of DLC resulted in the superior capacity to entrap more drugs in the micelle core assembled by the hydrophobic PCL and PDEAEMA blocks.

**Figure 6:**
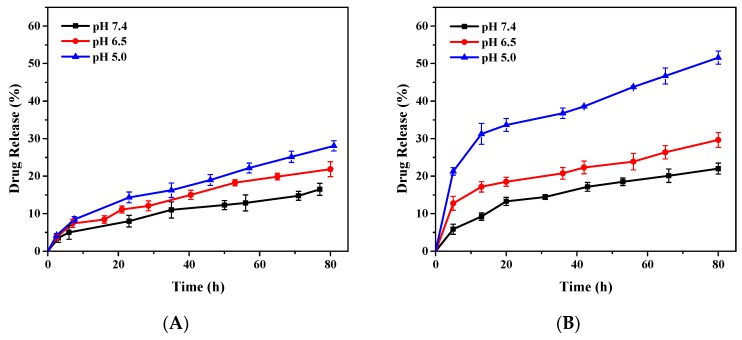
In vitro PTX release profiles of drug-loaded micelles (**A**) CA-CP and (**B**) CA-CDEP at 37 °C with different pH.

The presence of the PDEAEMA moiety in the CA-CDEP confers to the delivery system a responsiveness to acid. In vitro release of PTX at 37 °C was conducted at three different pH values. The results shown in Figure 6 compare the drug release of two micelles at three pH values. For CA-CP (Figure 6A), a less pronounced release of PTX was observed, and there was no significant increase in drug release during the gradual neutral to acidic phase of pH. In the environment of pH 5.0, the cumulative release of the drug at 80 h was 15% higher than that in the pH 7.4 environment. It can be seen in Figure 6B that the drug cumulative release of CA-CDEP micelles at pH 7.4 was about 20% at 80 h, avoiding the burst release of PTX during the blood circulation. However, when the pH decreased from 7.4 to 5.0, the cumulative release increased from 20% to 55%, which revealed well the pH-responsive drug release behavior of PTX-loaded CA-CDEP micelles.

The drug release from a polymeric system is a complex and varied process. A comprehensive, simple semi-empirical equation established by Peppas and co-workers is widely used to briefly analyze the release mechanism, called the power law [43]:

(4) $$\frac{M_{t}}{M_{\infty }}=kt^{n},$$

where *M_t_* and *M_∞_* are the absolute cumulative amount of drug released at time *t* and infinite time, respectively; *k* is a constant incorporating structural and geometric characteristics of the device; and *n* is the release exponent indicating the release mechanism. The value of *n* is determined by geometric configuration of the polymeric system. For a sphere, the value of *n* is equal to 0.43 for Fickian diffusion and 0.85 for case II transport, 0.43 < *n* < 0.85 belongs to anomalous transport mechanisms (non-Fickian diffusion), and *n* < 0.43 belongs to the erosion–diffusion common mechanism [44,45,46,47].

Figure S8 displays the power law curve of PTX release, and the related parameters are shown in Table 3 (R^2^ was the correlation coefficient). For CA-CP micelles, in the cases shown in Table 3 and Figure S8A, the PTX released mainly by an anomalous transport mechanism and all *n* values are in the range of 0.43–0.85. For CA-CDEP micelles, as shown in Table 3 and Figure S8B, at pH 7.4, the drug release was also mainly controlled by an anomalous transport mechanism due to the similar structure as CA-CP without the protonation of PDEAEMA. After the protonation of DEAEMA, the micelles were swelled and even dissociated at pH 6.5 and 5.0. PTX released from the CA-CDEP micelles was primarily controlled by a combination of diffusion and erosion control, and the *n* values were lower than 0.43. The *k* values under acidic conditions were higher than that of pH 7.4, suggesting the drug release rates accelerated as pH decreased. Moreover, the *k* values of CA-CDEP micelles were higher than that of CA-CP micelles at each pH condition due to the presence of PDEAEMA block in CA-CDEP.

Briefly, the drug release rate was significantly accelerated when changing pH from 7.4 to 5.0. The PTX release transformed from an anomalous transport mechanism to a joint control of diffusion and erosion caused by the presence of PDEAEMA. Drug was released from CA-CDEP micelles mainly through diffusion and erosion in an acidic environment.

### 3.4. In Vitro Cytotoxicity

The toxic side effects caused by the copper catalyst during polymerization were excluded by the XPS. As shown in Figure S9, for both two polymers, no copper element was detected, indicating the catalyst had been removed completely in the subsequent operation.

**Figure 7:**
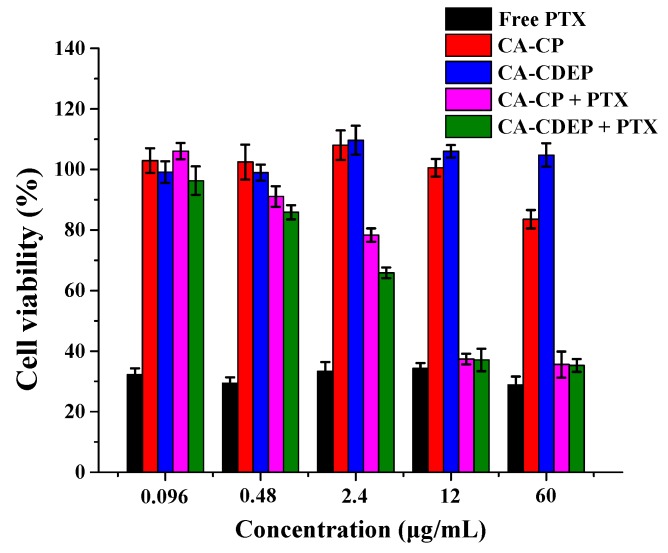
Cytotoxicity assay of the polymer blank micelles and drug-loaded micelles after 48 h incubation at different concentrations of polymer against NIH-3T3 cells using CCK-8 assay.

The toxicity of the blank micelles and the PTX-loaded micelles (5/10) was tested with NIH-3T3 cells, and the results are presented in Figure 7. While the concentration of blank micelles of CA-CP and CA-CDEP reached 60 μg/mL, the cell viability for both was over 80%. This demonstrated that the two blank micelles had great biocompatibility and were essentially non-toxic to NIH-3T3 cells at concentrations below 60 μg/mL. As shown in Figure S10, the IC_50_ of CA-CP blank micelles was 121.9 μg/mL, and no IC_50_ of CA-CDEP blank micelles was observed at the concentration tested, which showed a better biocompatibility than CA-CP blank micelles.

Cytotoxicity of the micelles after PTX loading was also evaluated. Interestingly, the cell viability of NIH-3T3 cells after 48 h incubation with culture medium containing drug-loaded micelles (12 μg/mL) dropped sharply. It was inferred that the phenomenon was caused by the concentration of PTX released from the drug-loaded micelles (12 μg/mL) in the medium, calculated combined with the Figure 6, was higher than the lowest concentration of free PTX (0.096 μg/mL). Therefore, the cell viability of two kinds of drug-loaded micelles (12 μg/mL), which was 38% for PTX-loaded CA-CP and 37% for PTX-loaded CA-CDEP, was still higher than that of the free PTX (0.096 μg/mL, 33% cell viability).

## 4. Conclusions

Two three-armed amphiphilic block polymers with a cholic acid core were designed and synthesized through a combination of ROP and ATRP. The CMC values of two polymers were relatively low and the assembled micelles in aqueous solution had a stable core-shell structure. The particle sizes of the micelles after PTX loading were both approximately 260 nm and the PTX loading of CA-CDEP was higher than that of CA-CP, with a maximum loading of 29.92 wt %. When the pH dropped from 7.4 to 5, the presence of PDEAEMA block increased the PTX release from 20% to 55%. The drug loading system can be used to alleviate the side effect of burst release during PTX administration. These polymers had expected biocompatibility to NIH-3T3 cells at a concentration below 60 μg/mL. More importantly, the issues regarding in vivo studies such as cytotoxicity, release, and further biocompatibility, etc., still need to be refined to achieve a complete drug controlled-release system. Undoubtedly, this drug controlled-release system based on cholic acid has great potential in the field of biomedical science. We anticipate that this work will provide more inspiration in the future for a drug delivery system based on cholic acid series.

## Figures and Tables

**Table 1:** Molecular weight measured by GPC and compared to the theoretical value.

Polymer	M_n,th_^1^ (g/mol)	M_n,GPC_ (g/mol)	PDI ^2^
CA-(PCL_28_)_3_	9500	9400	1.46
CA-(PCL_28_-*b*-PDEAEMA_5_)_3_	16,900	12,800	1.55
CA-CDEP	31,780	19,500	1.44
CA-CP	32,900	19,400	1.31

^1^ Value by design. ^2^ Polymer molecular weight dispersity indexes determined by GPC.

**Table 2:** Diameter, PDI, zeta potential, DLC, and EE of micelles.

Sample	PTX/Polymer	Diameter (nm)		PDI ^1^ (DLS)	Potential (Zeta, mV)	DLC (wt %)	EE (%)
		DLS	TEM				
CA-CP	--	151.0	100 ± 20	0.093	−14.96	--	--
CA-CDEP	--	170.4	120 ± 20	0.205	−16.04	--	--
CA-CP + PTX	30/100	245.2	--	0.147	--	6.96	22.17
	50/100	269.2	220 ± 20	0.105	--	11.4	23.14
CA-CDEP + PTX	30/100	230.6	--	0.097	--	20.48	41.77
	50/100	268.2	210 ± 20	0.171	--	29.92	48.24

^1^ Particle size dispersion index determined by DLS.

**Table 3:** Fitting parameters of PTX release data from PTX-loaded micelles at different pH.

Sample	pH	*n*	*k*	R^2^
CA-CP	7.4	0.45	0.11	0.991
	6.5	0.50	0.10	0.982
	5.0	0.52	0.10	0.993
CA-CDEP	7.4	0.47	0.11	0.986
	6.5	0.28	0.23	0.973
	5.0	0.29	0.22	0.967

## Supplementary Materials

The following are available online at https://www.mdpi.com/2073-4360/11/3/511/s1, Scheme S1: Synthesis of CA-(PCL_28_-*b*-PPEGMA_7_)_3_ (CA-CP), Figure S1: ^1^HNMR spectra of polymer CA-(PCL_28_)_3_, Figure S2: ^1^HNMR spectra of polymer CA-(PCL_28_-Br)_3_, Figure S3: ^1^HNMR spectra of polymer CA-(PCL_28_-PDEAEMA_5_-Br)_3_, Figure S4: ^1^HNMR spectra of CA-CP (red) and CA-CDEP (black), Figure S5: The pH-profile of CA-CDEP and CA-CP and NaCl by acid–base titration with 0.1 mol/L HCl and 0.1 mol/L NaOH, Figure S6: Effect of pH on the particle of the polymer micelles, Figure S7: Effect of pH on the zeta potential of the polymer micelles, Figure S8: Plots of (*M_t_*/*M*_∞_) against *t* for PTX release from PTX-loaded micelles at different pH, Figure S9: XPS spectra of the polymer CA-CDEP and CA-CP, Figure S10: IC_50_ concentration of free PTX, CA-CP, CA-CDEP, CA-CP + PTX, CA-CDEP + PTX.
