# Supplementary material for: pH-Responsive Micelles Assembled by Three-Armed Degradable Block Copolymers with a Cholic Acid Core for Drug Controlled-Release
Source: Polymers (Basel). 2019 Mar 18;11(3):511. doi: 10.3390/polym11030511 (PMC6473676; doi:10.3390/polym11030511)
Supplement: Supplementary file 1 [file polymers-11-00511-s001.pdf]

Supplementary Materials:

pH-Responsive Micelles Assembled by Three-Armed Degradable Block Copolymers with a Cholic Acid Core for Drug Controlled-Release

Jingjie Feng [1], Weiqiu Wen [1], Yong-Guang Jia [2], Sa Liu [2,*], and Jianwei Guo [1,*]

[1] School of Chemical Engineering & Light Industry, Guangdong University of Technology, Guangzhou 510006, China; fjj121036@163.com (J.F.); 15024028760@163.com (W.W.)

[2] School of Materials Science and Engineering, South China University of Technology, Guangzhou 510641, China; ygjia@scut.edu.cn (Y.-G.J.)

* Correspondence: guojw@gdut.edu.cn (J.G.); sliu@scut.edu.cn (S.L.); Tel: +86-20-3932-2232 (J.G.); +86-20-2223-6528 (S.L.)

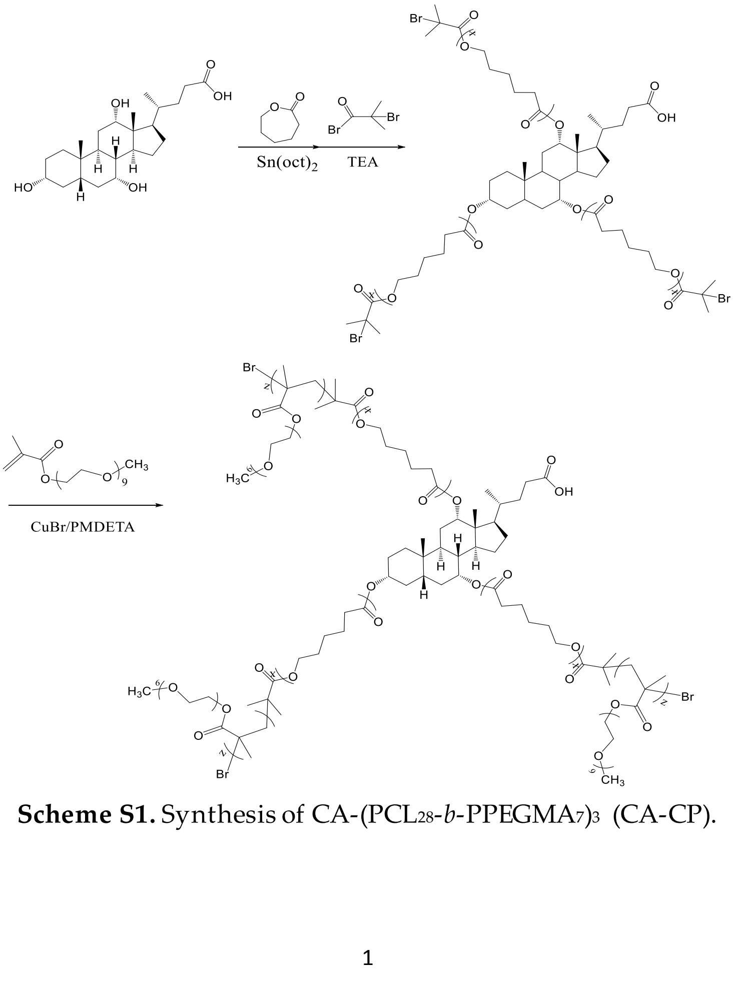

Scheme S1. Synthesis of CA-(PCL$_{28}$-*b*-PPEGMA$_{7}$)$_{3}$ (CA-CP).

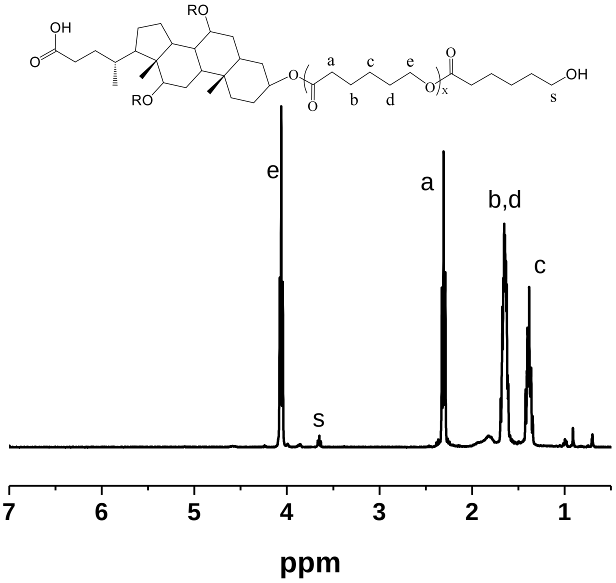

Figure S1. ¹HNMR spectra of polymer CA-$(PCL_{28})_3$.

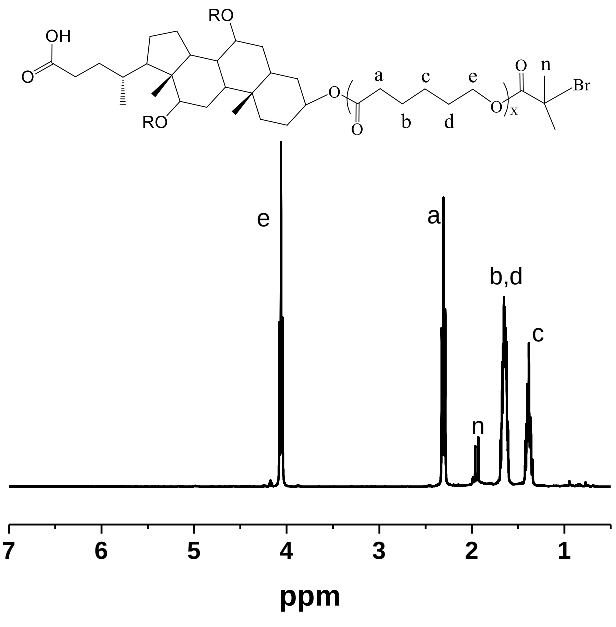

Figure S2. ¹HNMR spectra of polymer CA-$(PCL_{28}$-Br$)_3$.

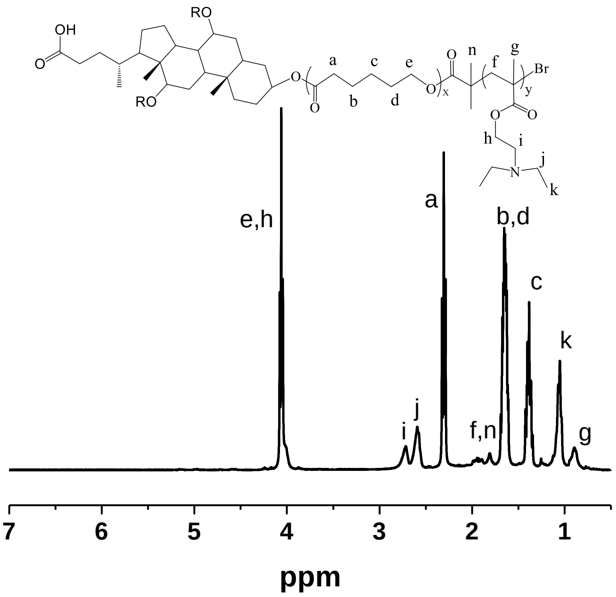

Figure S3. ^{1}HNMR spectra of polymer CA-(PCL_{28}-$PDEAEMA_{5}$-Br)$_3$.

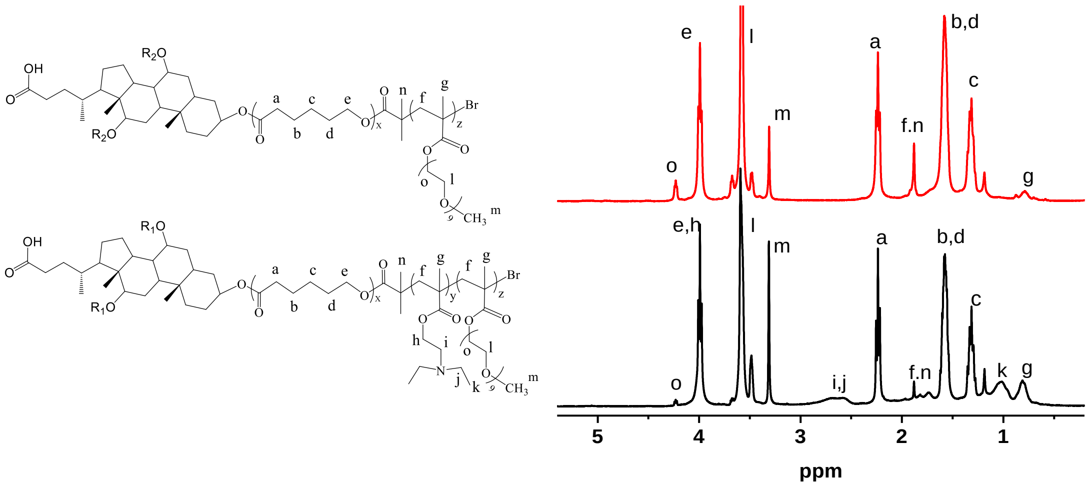

Figure S4. ^{1}HNMR spectra of CA-CP (red) and CA-CDEP (black).

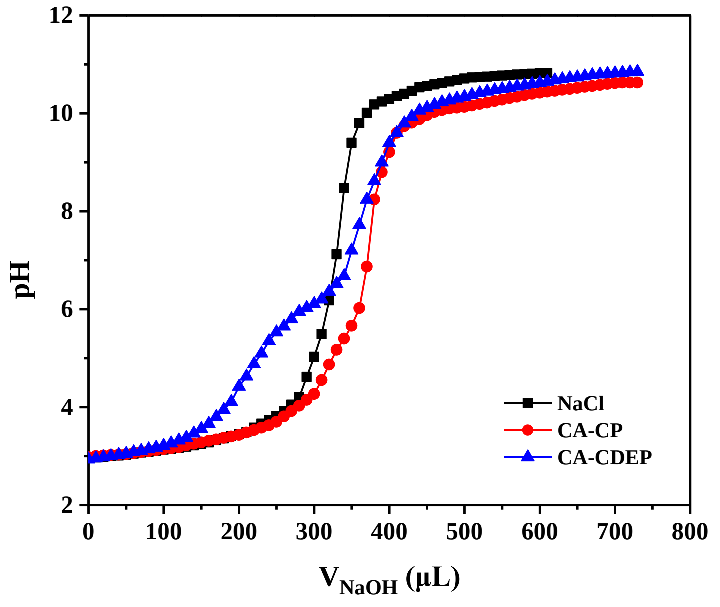

Figure S5. The pH-profile of CA-CDEP and CA-CP and NaCl by acid–base titration with 0.1 mol/L HCl and 0.1 mol/L NaOH.

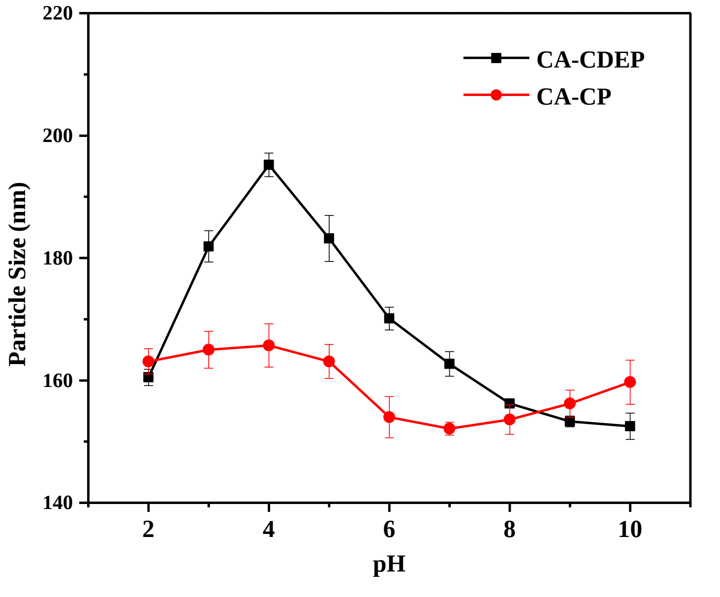

Figure S6. Effect of pH on the particle of the polymer micelles.

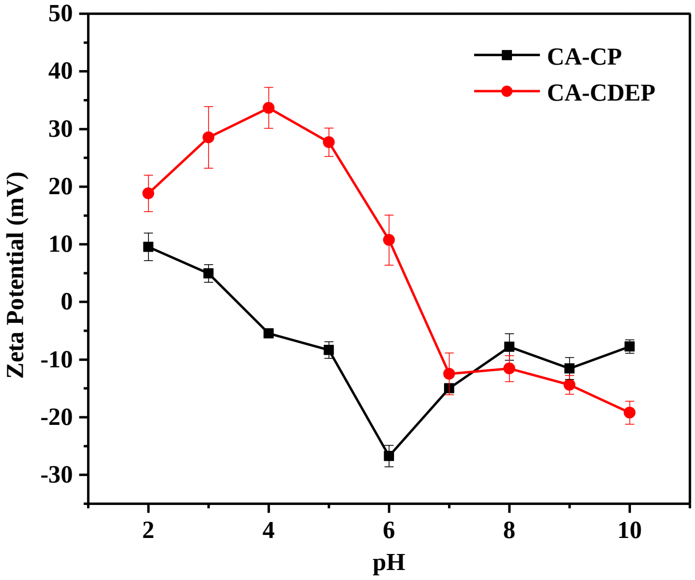

Figure S7. Effect of pH on the zeta potential of the polymer micelles.

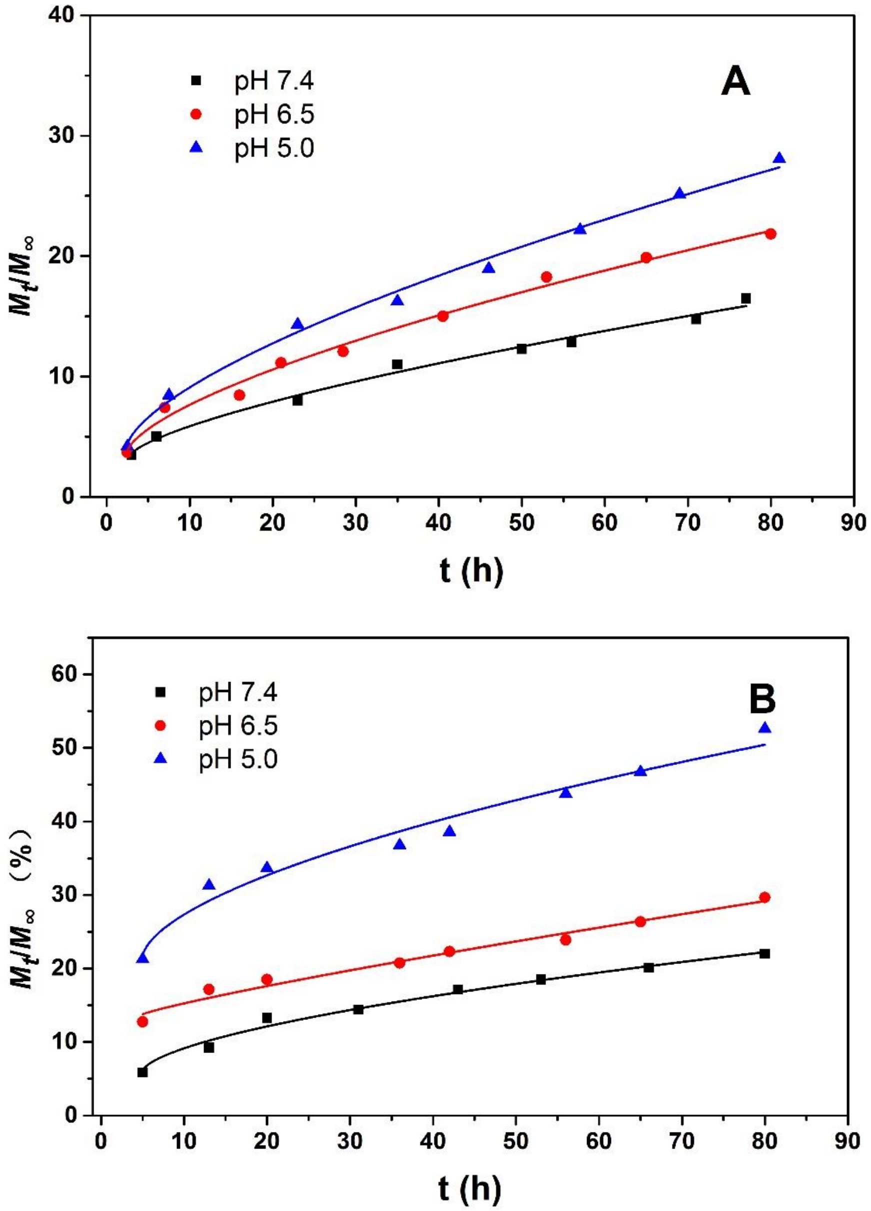

Figure S8. Plots of (M_t/M_∞) against t for PTX release from PTX-loaded micelles CA-CP (A) and CA-CDEP (B) at different pH.

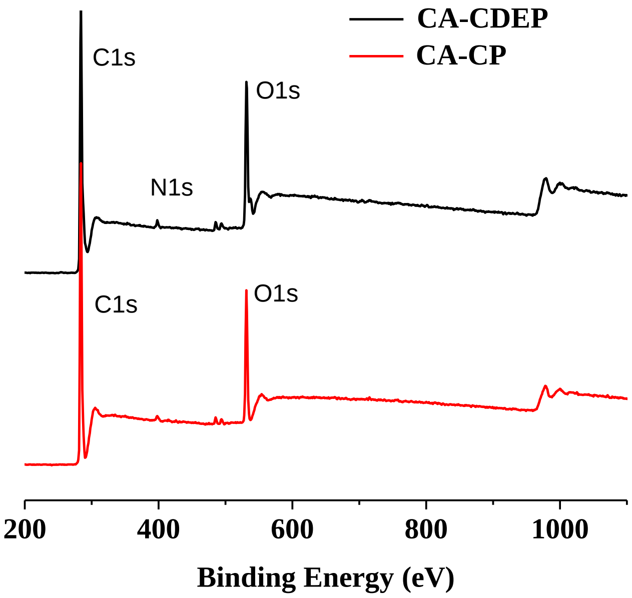

Figure S9. XPS spectra of the polymer CA-CDEP and CA-CP.

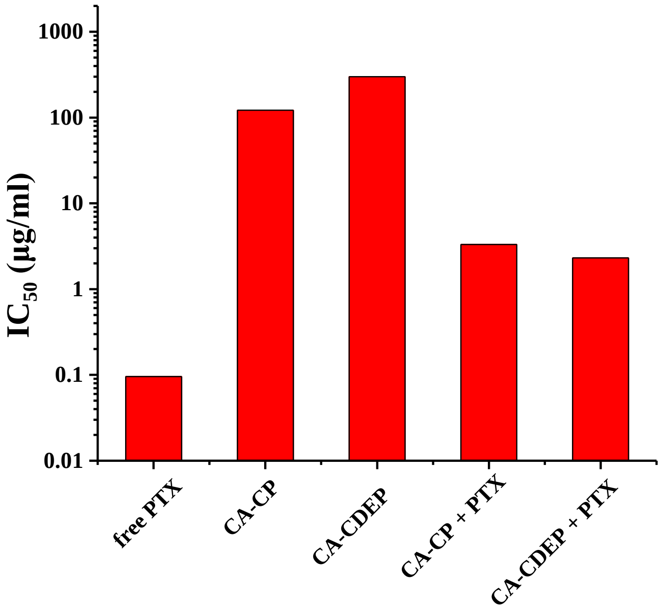

Figure S10. IC_{50} concentration of free PTX, CA-CP, CA-CDEP, CA-CP + PTX, CA-CDEP + PTX.
